# Research on the Influencing Factors of Problem-Driven Children’s Deep Learning

**DOI:** 10.3389/fpsyg.2022.764121

**Published:** 2022-04-12

**Authors:** Xiao-Hong Zhang, Chun-Yan Li

**Affiliations:** School of Education and Technonlogy, Shanxi Datong University, Datong, China

**Keywords:** children’s deep learning, problem-driven teaching, influencing factors, countermeasures, in-depth study

## Abstract

Deep learning is widely used in the fields of information technology and education innovation but there are few studies for young children in the preschool stage. Therefore, we aimed to explore factors that affect children’s learning ability through collecting relevant information from teachers in the kindergarten. Literature review, interview, and questionnaire survey methods were used to determine the influencing factors of deep learning. There were five dimensions for these factors: the level of difficulty of academic, communication skills, level of active collaboration, level of in-depth processing, and reflection level evaluation. Reliability and validity tests were used to analyze the data from questionnaires. In total, 100 valid questionnaires were collected. The Cronbach coefficients for academic challenge, communication, active cooperation, deep processing, and reflective evaluation were 0.801, 0.689, 0.770, 0.758, and 0.665, respectively. Principal component analysis revealed that there were three main factors that affect children’s learning depth: the level of deep processing (maximum KMO: 0.908), the level of reflective evaluation (maximum KMO: 0.542), and the active level of collaboration (maximum KMO: 0.410). In conclusion, there were several factors affecting deep learning in children and further studies are warranted to promote the development of this field.

**Graphical Abstract:**
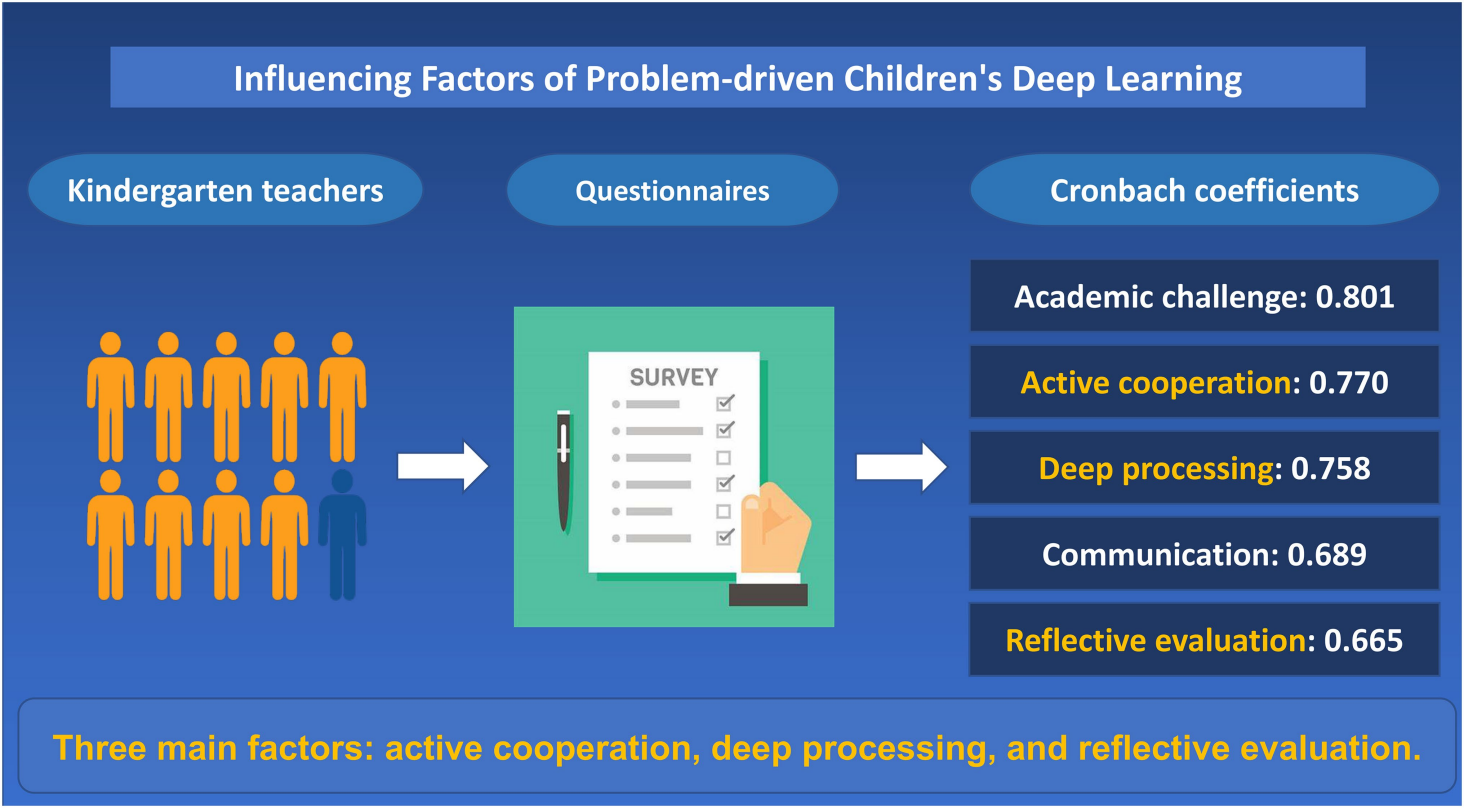
Investigation on the influencing factors of problem-driven children's deep learning.

## Introduction

High level of thinking and problem-solving ability can be cultivated through learning and was helpful in completing the work (D’Agostini et al., 2020; Xu et al., 2020), not just simple memory knowledge. Deep learning aims to make people think and act(Mingcheng, 2019). For children in the fast-growing period, deep learning is close to the effective scope of children’s recent development areas and is also an effective way to achieve meaningful learning (Zhang, 2020). Deep learning for children is a kind of personal experience, which can be transformed into a process beyond the original experience under the appropriate questions of the teacher, and promotes the development of individuals to a higher level (Linhares et al., 2019; Kroelinger et al., 2020).

Deep learning originated in the mid-1950s when American scholars Ference Marton and Roger Saljo jointly published “The Essential Difference of Learning: Results and Process” (Marton and Saljo, 1976). In 2005, Chinese scholars Professor Li Jiahou put forward the concept of deep learning for the first time(Nelson Laird et al., 2006). At the same time, other scholars put forward the characteristics of deep learning: learners need emotional input, pay attention to the exploration of the learning process, pay attention to the critical understanding of knowledge, and emphasize the connection between new and old knowledge (Li and Zu, 2020). Yoshua Bengio builds a deep learning method in machine learning based on feature extraction and feature selection in the emotional modeling process(Martinez et al., 2013). Use “deep learning” as the subject term to search and read and summarize key documents(Martinez et al., 2013). The present study(Terrenghi et al., 2019) aims to explore whether the “Episodes of Situated Learning” teaching methodology, according to literature about the Flipped Class model, has a positive outcome on student engagement, focusing on its emotional, cognitive, and behavioral components. The study includes a classroom of 16 students and three teachers. Pre-post measures, including video-recording, systematic observation, and questionnaires, of both students and teachers were collected during the 8 months of experimentation. The results show that Episodes of Situated Learning method can improve students’ interest in learning and bring positive influence to students.

In recent years, Chinese scholars have carried out future-oriented teaching reforms based on different subject backgrounds. Promoting students’ deep learning with in-depth teaching requires handling the multiple relationships between teachers and students, teaching environment and media, teaching strategies, and subject teaching (Fengfang, 2019).

Children’s deep learning means that teachers take the things that children often contact and are full of interest as the starting point of teaching, create challenging learning topics, and children take the initiative to think. It is a meaningful learning process to discover and proactively raise problems, transfer existing experience, and finally solve practical problems in life (Wang and Liu, 2020).

Nowadays, deep learning is widely used in the fields of information technology and education reform and innovation (Jiayang, 2019), but in the preschool stage, there are few studies on deep learning for young children. There are also few studies on the factors influencing children’s deep learning. Deep learning can arouse children’s active thinking, improve their interest in learning, and improve their ability and initiative to solve problems. There are few studies on children’s deep learning. Therefore, this study aims to explore the positive impact of deep learning on children’s learning through literature review and collection of relevant information.

## Research Methods

### Literature Reviewed Method

This research uses Internet electronic resources to search literature, systematically combs and analyzes works, journals and master and doctoral dissertations on deep learning and problem-driven teaching, grasps the current situation of teaching research development, briefly summarize and draw lessons from previous research results to support this paper, and provide basic theory and research guidance.

### Interview Method

In this study, the interview method is used mainly to determine the influencing factors of deep learning. After the author summarizes the influencing factors through the literature, teachers are invited to supplement and delete the obtained factors, and analyze the content of the research topic from a subjective perspective.

### Questionnaire Survey Method

This research mainly uses the questionnaire survey method to collect the influencing factors of question-driven deep learning for young children. The research object of the questionnaire survey is on-the-job teachers of preschool education to ensure the objective authenticity and scientific objectivity of the survey results.

#### Dimensions of the Questionnaire

In order to ensure the scientificity of the questionnaire design and its applicability to China’s educational environment, this questionnaire survey was compiled with reference to the 2015 National College Student Learning Investment Survey in the United States and the articles of Li Zhihe, Liu Dan, Li Ning, Li Fenqin, Yang Yuxia, and others in the 2018 Modern Education Technology Journal (Li et al., 2018). The questionnaire structure includes two main research aspects, namely, the basic information of the survey objects and the influencing factors of children’s deep learning.

The criteria for the factors affecting children’s deep learning in this assessment are obtained by collecting deep learning theories and consulting domestic and foreign literature: academic challenge level, communication level, in-depth processing level, active cooperation level, and reflective evaluation level. There was a total of 15 sub-item question frames, among which the details of the influencing factors of deep learning for young children are as follows: Table 1.

**Table 1:** Details of the dimensions of the influencing factors of children’s deep learning.

Dimension	Sub-item
Academic challenge level	1. Can apply the learned knowledge to specific situations to solve practical problems
	2. Invest sufficient time in learning, and at the same time get a certain guarantee in learning efficiency and quality
	3. Actively supplement knowledge in other fields after completing routine learning tasks
	4. Through in-depth learning, you can challenge your original views on the problem and accept new ones at the same time
	5. Be able to trace the root of a problem and further internalize it in its own knowledge system
Communication level	1. Able to discuss the confusion encountered in the learning process with peers and teachers
	2. Get their feedback in time after communicating with peers and teachers
	3. Can often discuss and communicate with teachers and peers in spare time
Active cooperation level	1. Actively cooperate with teachers in classroom learning
	2. Cooperate with peers during activities
Deep processing level	1. Express abstract knowledge in visual ways such as pictures and numbers
	2. Use the teacher’s targeted questions to in-depth processing and sorting out the fragmented knowledge learned
	3. In the learning process, be able to deeply integrate knowledge in different fields
Reflective evaluation level	1. Reflect and self-evaluate your own learning process
	2. During the learning process, you will ask your peers or teachers to evaluate your own learning

#### Implementation of The Questionnaire

The scales used in this questionnaire are all calculation methods of Likert-style 5-point scale. A total of 102 questionnaires were distributed. After the survey and research were conducted under the specified time and conditions, invalid questionnaires were removed, and 100 valid questionnaires were finally obtained.

#### Reliability and Validity Test

##### Reliability Test

The questionnaire used to test the reliability of the test is to test the internal reliability. The commonly used internal reliability coefficient is the widely used Cronbach coefficient (Cronbach’s α; Wang et al., 2020). If it is above 0.8, the reliability of the questionnaire test is high; the reliability coefficient is above 0.7 and the questionnaire can be widely accepted; and if it is lower than 0.6, the reliability of the questionnaire is low and the test items need to be redesigned. The SPSS software is used to analyze the samples collected about the influencing factors of children’s deep learning. The Cronbach coefficients for academic challenge, communication, active cooperation, deep processing, and reflective evaluation were 0.801, 0.689, 0.770, 0.758, and 0.665, respectively (Table 2).

**Table 2:** Reliability statistics.

Name	Corresponding item	Coefficient α
Academic challenge level	5	0.801
Communication level	3	0.689
Active cooperation level	2	0.770
Deep processing level	3	0.758
Reflective evaluation level	2	0.665

From the data point of view, the reliability coefficients of the influencing factors of children’s deep learning in the questionnaire of this study are all above 0.6, and the lowest is 0.665 (if there are two items in a dimension, the coefficient is greater than 0.6, indicating better reliability). Therefore, the reliability and quality of these data are good, and the research data are true and reliable.

##### Validity Test

Validity refers to the degree of validity required to achieve the test results. In research, the KMO value is generally used to indicate the validity. If the KMO value is greater than 0.6, the validity can be widely accepted. Then, the validity factor test of the questionnaire will be carried out. The KMO value of the questionnaire is 0.876 (Table 3), which indicates that the validity of the questionnaire is very high and reflects the characteristics that need to be measured.

**Table 3:** KMO and Bartlett’s test.

KMO value		0.876
Bartlett’s sphericity test	Approximate Chi-square	1320.467
	*df*	55
	value of *p*	0.000

In short, the research on the influencing factors of children’s deep learning shows that the reliability and validity of the questionnaire are very good, and the reliability and validity test can meet all the specific requirements of this study. At the same time, it ensures the scientificity of the research and can analyze the relevant scientific and reasonable statistical data and questionnaire data.

#### Participants

The basic information of the questionnaire survey objects includes gender, time spent as a kindergarten teacher and the class they are in. It will play a supporting role in the subsequent analysis of the factors affecting children’s deep learning. The specific situation is shown in Table 4. From the data point of view, the proportion of female teachers in the survey questionnaire is 98%, and only 2% are male teachers; 66% of teachers have been employed for less than 1 year, and only 4% for more than 5 years; and the number of surveyed teachers in the top and middle classes are the same, accounting for 38%, and nursery classes account for 6%.

**Table 4:** Basic data analysis.

Gender	Male		Female	
Percentage	2%		98%	
Working time	Less than 1 year	1–3 years	3–5 years	More than 5 years
Percentage	66%	20%	10%	4%
Class	Nursery class	Junior class of kindergarten	Middle class of kindergarten	Senior class of kindergarten
Percentage	6%	18%	38%	38%

## Results

Principal component analysis is a multivariate statistical method that examines the correlation between multiple variables (Ye et al., 2018). In view of understanding the key factors of principal component analysis, this research aims to discover and determine the key factors that affect children’s deep learning through influencing factors. Whether it is suitable for principal component analysis depends on the analysis results of the key components of KMO and Bartlett. As shown in Table 5, the KMO value is 0.767, which is greater than 0.6, which meets the basic requirements of principal component analysis and can perform principal component analysis.

**Table 5:** KMO and Bartlett’s test.

KMO value		0.767
Bartlett’s sphericity test	Approximate Chi-square	335.553
	*df*	105
	value of *p*	0.000

In principle, when the key components are executed in the evaluation phase, the characteristic root should be used as the setting index of the test, but the main components of the analysis have been determined in this study. Therefore, the characteristic root greater than 1 is not used. The analysis results are shown in Tables 6, 7.

**Table 6:** Total variance explained by principal component analysis of influencing factors of deep learning.

Component	Initial eigenvalue			Extract sum of squares loading		
	Total	% of variance	% of accumulation	Total	% of variance	% of accumulation
1	3.734	74.690	74.690	3.734	74.690	74.690
2	0.442	8.837	83.527	0.442	8.837	83.527
3	0.323	6.476	90.003	0.323	6.476	90.003
4	0.280	5.615	95.608			
5	0.220	4.392	100.000			

**Table 7:** Principal component analysis coefficient matrix of factors influencing deep learning.

	Component		
	1	2	3
Academic challenge level	0.876	−0.062	−0.308
Communication level	0.862	−0.265	−0.218
Active cooperation level	0.855	−0.265	0.410
Deep processing level	0.908	0.075	0.062
Reflective evaluation level	0.810	0.542	0.053

From the data point of view, through the method of principal component extraction, three key components are extracted, which are the three most important factors that contribute to the deep learning of young children. According to the selection principle, the main components are the deep processing level (maximum 0.908), reflective evaluation level (maximum 0.542), and active cooperation level (maximum 0.410), explaining 90.003% of all variance. According to the total variance component score coefficient matrix explained by the principal component analysis in Table 6, we can get the common factor expression: F1 = 0.410X_3_ + 0.908X_4_ + 0.542X_5_.

## Discussion

In deep learning, learners need emotional input, pay attention to the exploration of the learning process, pay attention to the critical understanding of knowledge, and emphasize the connection between new and old knowledge(Li and Zu, 2020). Our results are consistent with this. According to the results of this questionnaire survey, the main factors affecting children’s deep learning are the level of active cooperation, the level of deep processing, and the level of reflective evaluation. The specific analysis is as follows:

**Active cooperation level:** Children can actively collaborate, solve problems, and integrate games, learning, and interpersonal relationships into their lives. At the same time, everyone has achieved the goal of mutual cooperation. But in fact, children lack both the awareness of cooperation and the ability to cooperate. Children’s conflicts in play are usually resolved by complaining and aggressive behavior, without asking for help, or consciously solving problems when encountering difficulties. The prerequisite for a person to succeed is to have good cooperation and communication skills (Andersson et al., 2020). Toddlers need to learn cooperative skills in order to learn more knowledge.

**In-depth processing level:** In deep learning, students participate in the learning as co-designers and co-learners, combining their internal development with the external world (MacKinnon et al., 2017). An important direction of the development of deep learning is to be able to complete the transfer of old and new experiences. The ability of young children to express their current experience and transfer their current experience to the current problematic situation is an important indicator of children’s in-depth learning. However, young children are not clear about the existing experience, nor do they have the ability to transfer knowledge. Knowledge transfer ability can improve children’s learning literacy, which is essential for deep learning.

**Reflective evaluation level:** In deep learning, children need to be able to effectively monitor their own behavior, flexibly master the activity time, and correctly reflect and analyze the results of their own activities. However, young children often do not reflect on their own behavior. The end of the event means the end of all. They will not think deeply, or review their behavior in the activity, and what they do inappropriately will still be inappropriate in the next event. Without reflection and evaluation, people will not be able to realize their own problems, which is very detrimental to their future study and life.

Young children’s deep learning ability can be improved by the following methods: Flexible use of problem scenarios to improve children’s active cooperation level (Xu et al., 2020): Cooperation and communication skills are one of the necessary skills for human survival, and it is also a prerequisite for children’s better development in the future. In order to efficiently cultivate children’s communication and cooperation skills, it is not enough to cultivate children’s cooperative spirit. It is also necessary to cultivate children’s practical cooperation ability so that children can fully master cooperation skills and learn more knowledge in cooperative behavior. Children should help each other and benefit each other. At the same time, the partnership between teachers and children is also very important. In general, teachers play three roles: activator, cultural builder, and collaborator in the learning partnership (Full An et al., 2018). Situational teaching will help to improve and deepen children’s cooperation ability. Teachers can create some cooperative situations for children at any time, and children can carry out cooperative behaviors in this situation. Flexible use of problem scenarios to improve children’s in-depth processing level (D’Agostini et al., 2020): Children’s deep learning means that children learn new knowledge, gain new experiences, connect with the natural environment and social environment, and learn from the environment. Therefore, gaining knowledge and experiences is an important part of children’s in-depth learning. Children extract experience in the process of inquiry, incorporate these knowledge and experiences into their own original experience system, and complete the migration and integration of new and old experiences. This kind of learning is meaningful. Throughout the teaching process, the teacher must help the students to systemize and integrate the knowledge they have learned, and guide the students to complete the common transfer of the same or similar knowledge (Söderback and Frost, 1995). It is necessary to rationally use the problem situation, ask targeted questions, extract key experiences, and guide children to transfer knowledge. Flexible use of problem situations to improve children’s reflection and evaluation level (Mingcheng, 2019): Based on the theory of situational cognition, starting with the problems at the current stage, in-depth application of the situation, flexible use of the information contained in the situation to guide children to conduct in-depth learning, in order to enhance the challenge and inquiry of practical problems, and improve the efficiency of knowledge utilization. At the same time, the social environment and the natural environment are combined to construct a good situational model, which is related to life and creates a high-quality environment. Create activities based on the actual situation, closer to the reality of life, so that children can understand the transfer of knowledge and follow deep learning. In order to improve the level and quality of child-centered thinking, guide children to learn independent thinking and standardize review and reflection behavior. Reflective assessment is the foundation of children’s learning awareness and the key force to promote children’s learning. Instruct children to review their learning status in different activities, review and reflect on their own words and activities, and then make self-adjustment and correction, thereby improving the effectiveness of learning.

## Conclusion

This research explores the influencing factors of children’s deep learning. First, through literature reviewed methods to determine the influencing factors of children’s deep learning, and get five dimensions that affect children’s deep learning. Then, a questionnaire survey and online interviews are conducted with kindergarten teachers. Finally, according to the survey results, the main factors that affect children’s deep learning are obtained, and corresponding opinions and suggestions are put forward.

During the development and implementation of this research, due to the influence and constraints of various realistic conditions, the research still has the following limitations:

The research objects of this study are required to be kindergarten teachers, and only one kindergarten is selected for the survey when selecting the sample, so there is no comparison in the research objects. The theoretical support for deep learning for young children is relatively weak, and there are few related documents. The research on deep learning for young children is still in the preliminary exploration stage and is not yet mature. Future research can continue to develop and improve this survey, and promote the development of children’s deep learning.

## Data Availability Statement

The original contributions presented in the study are included in the article/supplementary material, further inquiries can be directed to the corresponding author.

## Ethics Statement

The studies involving human participants were reviewed and approved by Ethics committee of Shanxi Datong University. Written informed consent to participate in this study was provided by the participants’ legal guardian/next of kin.

## Author Contributions

X-HZ: guarantor of integrity of the entire study, study concept and design, literature research, manuscript preparation, and manuscript editing. C-YL: data analysis and statistical analysis. X-HZ and C-YL contributed to the article and approved the submitted version.

## Funding

This work was supported by Shanxi Province Educational Science Planning Leading Group Office, Research on the construction of education quality system for inclusive private kindergartens (GH-19058) and Philosophy and Social Science Project of Shanxi Provincial Education Department, Research on the quality monitoring system of preschool education in rural areas of Shanxi Province (2019W110).

## Conflict of Interest

The authors declare that the research was conducted in the absence of any commercial or financial relationships that could be construed as a potential conflict of interest.

## Publisher’s Note

All claims expressed in this article are solely those of the authors and do not necessarily represent those of their affiliated organizations, or those of the publisher, the editors and the reviewers. Any product that may be evaluated in this article, or claim that may be made by its manufacturer, is not guaranteed or endorsed by the publisher.

## References

[ref1] Andersson K. P. Chang K. Molina-Garzón A. (2020). Voluntary leadership and the emergence of institutions for self-governance. Proc. Natl. Acad. Sci. U. S. A. 117, 27292–27299. doi: 10.1073/pnas.2007230117, PMID: 33067395 PMC7959538

[ref2] D’Agostini M. M. Aredes N. D. A. Campbell S. H. Fonseca L. M. M. (2020). Serious game e-baby família: an educational technology for premature infant care. Rev. Bras. Enferm. 73:e20190116. doi: 10.1590/0034-7167-2019-0116 32609174

[ref3] Fengfang S. (2019). The teaching strategy of promoting students’ deep learning. Fujian Edu. Res. 3, 95–96.

[ref4] Full An M. Quinn J. Mceachen J. (2018). Deep Learning: Engage the World Change the World. Thousand Oaks, CA: Corwin.

[ref5] Jiayang X. (2019). Research on the path of education reform for deep learning. Good Parents 47:1.

[ref6] Kroelinger C. D. Addison D. Rodriguez M. Rice M. E. Frey M. T. Hickner H. R.. (2020). Implementing a learning collaborative framework for states working to improve outcomes for vulnerable populations: The opioid use disorder, maternal outcomes, and neonatal abstinence syndrome initiative learning community. J. Womens Health 29, 475–486. doi: 10.1089/jwh.2020.8303 PMC7259818 32176568

[ref7] Li Z. H. Liu D. Li N. (2018). A study on the influencing factors of deep learning under the flipped classroom model. Mod. Educ. Technol. 28, 55–61.

[ref8] Li C. L. Zu J. (2020). Exploration based on the inducing conditions of children's deep learning. Liaoning Edu. 8, 55–58.

[ref9] Linhares E. F. Dias J. A. A. Santos M. D. C. Q. D. Boery R. N. S. O. Santos N. A. FEF M. (2019). Collective memory of umbilical cord stump care: an educational experience. Rev. Bras. Enferm. 72, 360–364. doi: 10.1590/0034-7167-2018-0735, PMID: 31851274

[ref10] MacKinnon K. Marcellus L. Rivers J. Gordon C. Ryan M. Butcher D. (2017). Student and educator experiences of maternal-child simulation-based learning: a systematic review of qualitative evidence. JBI Data. System Rev. Impl. Rep. 15, 2666–2706. doi: 10.11124/JBISRIR-2016-003147, PMID: 29135750

[ref11] Martinez H. P. Bengio Y. Yannakakis G. N. (2013). Learning deep physiological models of affect. IEEE Comp. Int. Mag. 8, 20–33. doi: 10.1109/MCI.2013.2247823

[ref12] Marton F. Saljo R. (1976). On qualitative difference in learning: outcome and process. J. Educ. Psychol. 46, 4–11. doi: 10.1111/j.2044-8279.1976.tb02980.x

[ref13] Mingcheng L. (2019). Towards deep learning: construction and implementation strategies of "thinking class". Prim. Sec. School Manage. 12, 39–41.

[ref14] Nelson Laird T. F. Shoup R. Kuh G. D. (2006). “Measuring deep approaches to learning using the National Survey of student engagement,” in *The Annual Forum of the Association for Institutional Research*; April 14, 2006.

[ref15] Söderback I. Frost D. (1995). The transfer of knowledge in occupational therapy: the case of work ability assessment. Work 5, 157–165. doi: 10.3233/WOR-1995-5302, PMID: 24441268

[ref16] Terrenghi I. Diana B. Zurloni V. Rivoltella P. C. Elia M. Castañer M.. (2019). Episode of situated learning to enhance student engagement and promote deep learning: preliminary results in a high school classroom. Front. Psychol. 10:1415. doi: 10.3389/fpsyg.2019.01415, PMID: 31297074 PMC6607896

[ref17] Wang H. Y. Chou W. Shao Y. Chien T. W. (2020). Comparison of Ferguson's δ and the Gini coefficient used for measuring the inequality of data related to health quality of life outcomes. Health Qual. Life Outcomes 18:111. doi: 10.1186/s12955-020-01356-6, PMID: 32345296 PMC7189694

[ref18] Wang X. Y. Liu S. Y. (2020). The basic characteristics and logical structure of children's deep learning. Pre. Edu. Res. 1, 3–10.

[ref19] Xu M. Liu A. Zhao C. Fang H. Huang X. Berman S.. (2020). Group-based intervention to improve developmental status among children age 6–18 months in rural Shanxi province, China: a study protocol for a cluster randomised controlled trial. BMJ Open 10:e037156. doi: 10.1136/bmjopen-2020-037156, PMID: 33077560 PMC7574939

[ref20] Ye F. Crippa G. Garbelli C. Griesshaber E. (2018). Microstructural data of six recent brachiopod species: SEM, EBSD, morphometric and statistical analyses. Data Brief 18, 300–318. doi: 10.1016/j.dib.2018.02.071, PMID: 29896518 PMC5995785

[ref21] Zhang B. (2020). Value examination, academic pursuit and practical exploration of children's deep Chinese learning. Chin. Constr. 2, 21–24.

